# Alternating‐contrast single‐shot spiral MR‐ARFI with model‐based displacement map reconstruction

**DOI:** 10.1002/mrm.70066

**Published:** 2025-09-02

**Authors:** Saikat Sengupta, M. Anthony Phipps, Li Min Chen, Charles F. Caskey, William A. Grissom

**Affiliations:** ^1^ Department of Radiology Vanderbilt University Medical Center Nashville Tennessee USA; ^2^ Department of Biomedical Engineering Vanderbilt University Nashville Tennessee USA; ^3^ Department of Biomedical Engineering Case Western Reserve University Cleveland Ohio USA

**Keywords:** focused ultrasound, MR‐ARFI, neuroimaging, neuromodulation, spiral

## Abstract

**Purpose:**

To improve single‐shot spiral MR‐Acoustic Radiation Force Imaging (MR‐ARFI)'s robustness to dynamic phase errors and evaluate it in non‐human primates (NHPs) with a low‐f‐number transducer.

**Methods:**

A single‐shot spiral MR‐ARFI pulse sequence with 2 mm in‐plane resolution and alternating displacement phase contrast was implemented to visualize the focus generated by a 128‐element ultrasound transducer in the NHP brain. A model‐based displacement map calculation was implemented to remove dynamic phase errors. MR‐ARFI scans were acquired at pressure levels above and below FDA mechanical index (MI) limits, and reconstructed displacement maps were compared to maps generated by a 3D EPI MR‐ARFI scan and a spiral MR‐ARFI scan with blocked ultrasound triggering.

**Results:**

The proposed sequence and processing detected focal tissue displacements of 160 nm at a transcranial mechanical index of 0.96, which the 3D EPI could not detect, and with 9.7×‐improved precision. The model‐based reconstruction suppressed background phase errors and maximized precision. Alternating contrast yielded displacement maps with 4.9×‐improved precision compared to blocked contrast.

**Conclusion:**

Single‐shot spiral MR‐ARFI can provide robust focus visualization in MR‐guided ultrasound in the brain at MI levels well below the FDA limit.

## 1 INTRODUCTION

Transcranial ultrasound stimulation (TUS) is an emerging non‐invasive neuromodulation modality with the ability to target deep brain regions with millimeter‐level precision,^1^ whose set of potential therapeutic applications is expanding rapidly.^2^, ^3^, ^4^, ^5^, ^6^, ^7^, ^8^ However, the high and variable acoustic attenuation of skull bone causes subject‐dependent distortions and spatial shifts of the ultrasound focus, so that the precise application of TUS benefits from the ability to visualize where the focus actually appears in the brain and re‐steer it to its intended location.^9^ Magnetic resonance acoustic radiation force imaging (MR‐ARFI)^10^, ^11^ is the most promising method to visualize a TUS focus without tissue heating.^12^ MR‐ARFI encodes the tissue displacement generated by an ultrasound pulse into the image phase by applying an ultrasound pulse simultaneously with a motion encoding gradient (MEG) applied along the ultrasound's propagation direction. A displacement map is calculated from the difference of phase images acquired with positive and negative displacement phase contrast.

In practice, MR‐ARFI's sensitivity is limited by the submicron‐level focal displacements achievable within thermal and mechanical safety limits.^12^ This leads to small focal phase shifts, less than a tenth of a radian in amplitude that are easily overwhelmed by ghosting and physiological phase artifacts in conventional 2DFT and EPI MR‐ARFI scans. To alleviate this sensitivity, Mohammadjavadi et al. recently proposed a highly averaged single‐shot spiral MR‐ARFI sequence.^13^ Using fMRI‐like processing to remove physiological phase artifacts and generate a t‐score map of significant displacements, the authors demonstrated successful in vivo MR‐ARFI with a high‐f‐number transducer with derated pressures below 1 MPa. To facilitate block design analyses, the authors collected the two MR‐ARFI phase contrasts in on‐off blocks.

This Technical Note proposes single‐shot spiral MR‐ARFI with alternating every‐other‐time point phase contrast rather than a block design, to minimize the average time between phase contrasts and better “freeze” dynamic physiological effects. We further describe a model‐based displacement map calculation algorithm that maximizes the benefit of averaging and produces quantitative displacement maps. Using a low‐f‐number transducer^14^ which produces lower displacement at a given pressure level than a high‐f‐number transducer,^15^ non‐human primate (NHP) MR‐ARFI data were collected and processed using the proposed sequence and algorithm across pressure levels. The resulting displacement maps were compared to a duration‐matched 3D EPI MR‐ARFI sequence that is currently used for TUS focus steering in NHP neuromodulation studies but with above‐limit pressure levels that preclude human application,^9^ and to block‐design spiral MR‐ARFI.

## 2 METHODS

### 2.1 Pulse sequence

**FIGURE 1:**
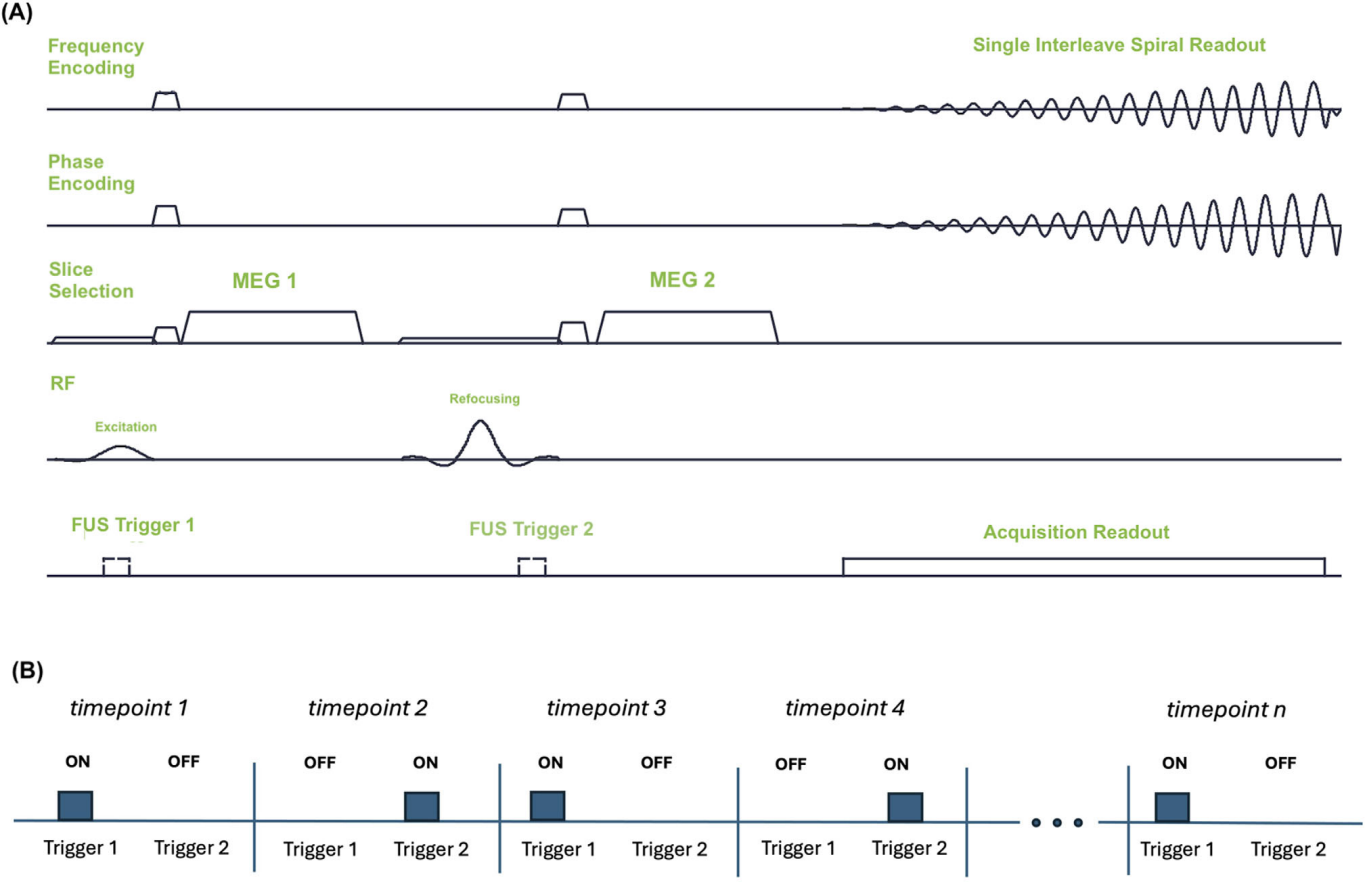
(A) The imaging sequence was a spin echo with a single‐shot spiral readout and with ARFI motion encoding gradients (MEGs) placed along the slice selection axis. The ultrasound (FUS) was triggered in advance of the MEGs to maximize the average displacement during the MEGs. (B) The ultrasound trigger was switched between MEGs every image repetition/time point. Since each consecutive pair of images comprises both phase contrasts, each pair was considered an average.

Figure 1A shows the spiral MR‐ARFI pulse sequence, which was implemented on a 3 Tesla human MRI scanner (Philips Elition, Philips Healthcare, Best, Netherlands). The sequence used unipolar, 40 mT/m, 7‐ms‐long MEGs spaced 9 ms apart. The MEGs were applied in the slice dimension to encode displacement in a plane parallel to the transducer face, and remained fixed for the entire scan. The ultrasound triggers preceded the MEGs by 3 ms to maximize average displacement during the MEGs. As further illustrated in Figure 1B, the triggers were alternated between the two MEGs every other image repetition/timepoint, so that a complete “average” was collected every two timepoints. This strategy was chosen to minimize the time between measuring the two phase contrasts, in contrast to Reference [13] which collected positive and negative displacement phase contrast in longer blocks. The sequence also used crusher gradients on all three axes around the refocusing pulse. The readout was a variable density single‐interleave spiral with duration 27.5 ms that collected data for a 160×160 mm^2^ FOV with 2×2 mm^2^ voxel size, which were reconstructed to 1×1 mm^2^ voxel size. The sequence used TR/TE of 1000/30 ms and 4 mm slice thickness. It was run in single‐slice and six‐slice modes, where the slice loop was innermost, and the volume TR was equal to the number of slices times 1000 ms. The total scan duration was proportional to the volume TR and the number of timepoints acquired.

### 2.2 ARFI displacement map calculation

**FIGURE 2:**
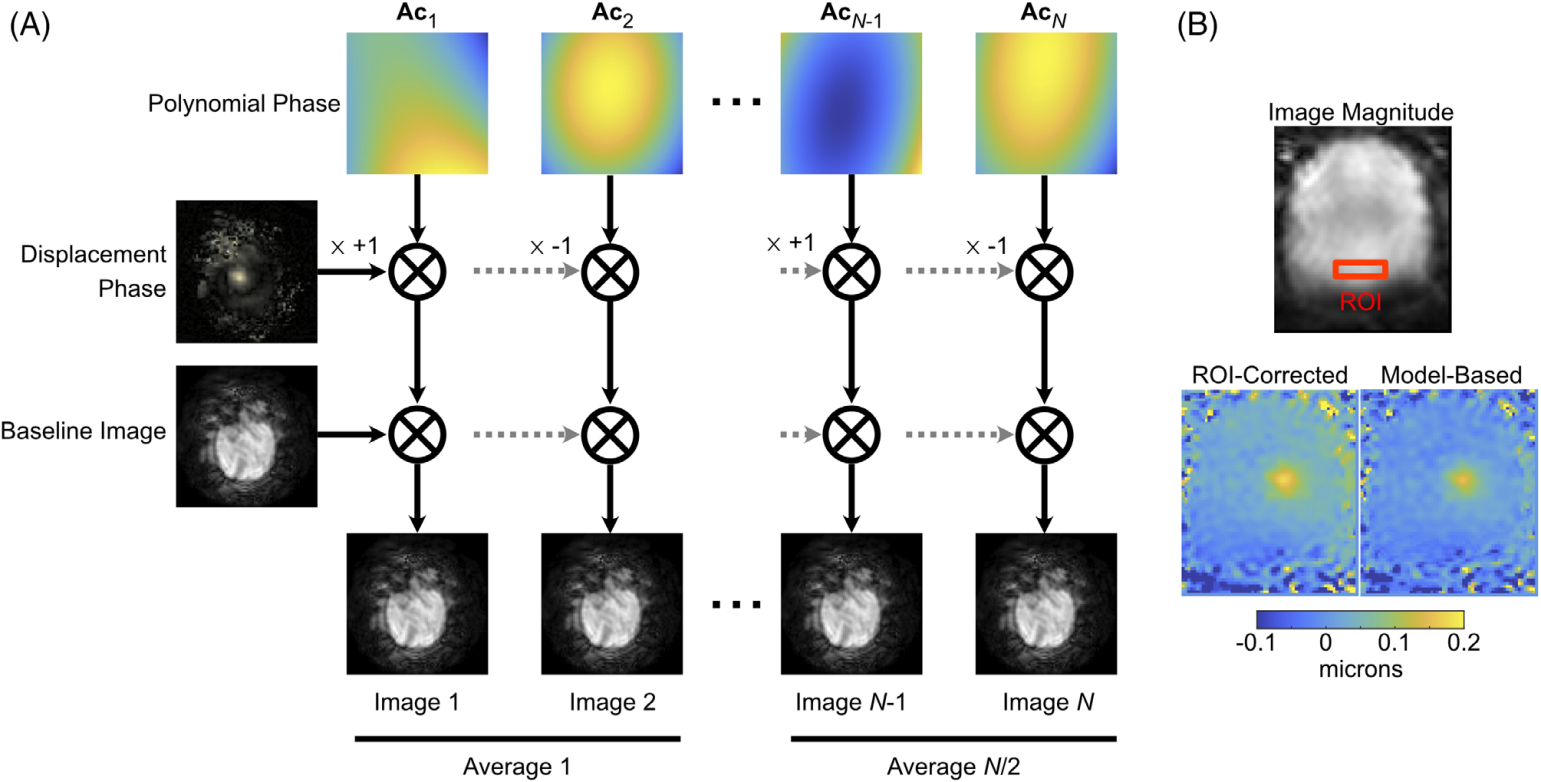
(A) Model for MR‐ARFI displacement map calculation. Each image is modeled as the product of a polynomial phase shift whose coefficients are estimated uniquely for that time point, a common displacement phase shift which is negated every other image as the phase contrast is inverted by switching the trigger between MEGs, and a common complex‐valued baseline image. (B) Example of ROI‐corrected vs. model‐based displacement maps (0.8 MPa). ROI‐corrected requires selection of the phase correction ROI, which is a 10×20 mm^2^ box indicated on the magnitude image. Compared to the ROI‐corrected map, the model‐based calculation removes a background displacement gradient and offset of approximately 30 nanometers, and attenuates erroneous displacements outside the brain.

Displacement maps were calculated from the complex‐valued images collected for each slice and time point. Figure 2 illustrates the model that was fit to the images, which is based on a hybrid multibaseline subtraction and referenceless model for MRI temperature images^16^ and for the image at timepoint n is written as:

(1) $${Î}_{n}\left(x_{j};m,c_{n},\theta \right)=m_{j}e^{ı\left({\left\{{\mathrm{Ac}}_{n}\right\}}_{j}+{\left(-1\right)}^{1[n]}\theta_{j}\right)},$$

where n indexes image timepoints, j indexes space, m is a complex‐valued baseline image with no displacement phase contrast, A is a polynomial matrix, $c_{n}$ is a vector of polynomial coefficients for timepoint n, 1[n] is an indicator function that takes the value 1 if the phase contrast is negated at timepoint n, and 0 otherwise, and θ is the displacement phase at timepoint n. A second‐order polynomial was used in this work. The variables $\left(m,{\left\{c\right\}}_{n=1}^{N_{t}},\theta \right)$ were obtained using PyTorch's lbfgs() optimizer applied to minimize the loss function:

(2) $$\frac{1}{N_{t}}\overset{N_{t}}{\underset{n=1}{\sum }}\overset{N_{s}}{\underset{j=1}{\sum }}{\left|I_{n}\left(x_{j}\right)-{Î}_{n}\left(x_{j};m_{j},c_{n},\theta_{j}\right)\right|}^{2}+\lambda \overset{N_{s}}{\underset{j=1}{\sum }}\left|\theta_{j}\right|,$$

where λ is a regularization parameter that encourages θ to be sparse, which reflects the prior knowledge that the focal displacement phase shifts will occupy a minority of image voxels, and it is necessary to separate the focal displacement phase shifts from spatially smooth, non‐sparse phase shifts that are modeled by the polynomial. λ=2000 was used in this work, while the measured images had a mean amplitude of approximately $10^{4}$. lbfgs() directly optimized θ and ${\left\{c\right\}}_{n=1}^{N_{t}}$ while the following analytic solution for m was substituted at each loss function evaluation:

(3) $${\hat{m}}_{j}=\frac{1}{N_{t}}\overset{N_{t}}{\underset{n=1}{\sum }}I_{n}\left(x_{j}\right)e^{-ı\left({\left\{{\mathrm{Ac}}_{n}\right\}}_{j}+{\left(-1\right)}^{1[n]}\theta_{j}\right)},$$

which is equal to the mean of the images over time points, after removing the polynomial and displacement phase from each image. Once θ was obtained, the displacement in meters was calculated by dividing it by 2π42.58GT, where G is the MEG gradient strength in mT/m, and T is the MEG duration in ms. This displacement map calculation method was compared to a region‐of‐interest (ROI) phase‐corrected method implemented by subtracting the phase in a manually selected out‐of‐focus rectangular ROI from each image, calculating the mean ROI phase‐corrected positive and mean negative phase contrast images, and then calculating their phase difference as the displacement phase map.

### 2.3 Experiments

MR‐ARFI scans were performed in a healthy adult female macaque monkey weighing 4.2 kg. The scan protocol was approved by the Institutional Animal Care and Use Committee (IACUC) at Vanderbilt University Medical Center, and all procedures were in agreement with institutional guidelines and regulations. The overall physical experimental setup has been detailed previously.^9^ The animal was anesthetized with 1%–1.5% isoflurane and mechanically ventilated. Its head was positioned in an MRI‐compatible stereotactic frame in the sphinx position. A pair of loop receive coils (Philips dStream Flex‐S) was placed on either side of the head.

The transducer was positioned for a deep‐brain focus location, and the imaging FOV was aligned with the ultrasound focus.^9^ The imaging slices were oriented to face the transducer and were transverse with respect to the brain. Sonications were performed with a 128‐element randomized phased array with a radius of curvature of 72 mm and a diameter of 103 mm operated at 650 kHz, which had a free‐field pressure, full width at half maximum of 2.2 mm and 9.3 mm in the lateral and axial directions.^14^ 8.5‐ms‐long pulses were generated each time a trigger was received. The transducer was driven by a custom‐built amplifier (Image Guided Therapy, Pessac, France).

Experiments were performed to compare the spiral sequence with model‐based displacement map calculation to a previously described 3D spin echo EPI MR‐ARFI sequence.^9^ The 3D EPI MR‐ARFI sequence's triggering and MEG amplitudes and durations were matched to the spiral sequence. It was also matched with the spiral sequence in acquired in‐plane voxel and matrix sizes and slice thickness, and it used TR/TE of 500/35 ms. As described in Reference,^9^ the EPI data were collected with interleaved FUS on/off and MEG polarities as: FUS off/positive MEGs, FUS on/positive MEGs, FUS off/negative MEGs, FUS on/negative MEGs. Displacement maps were calculated from the difference of the difference between the FUS off and on images, for the two polarities.^12^ Both spiral and EPI scans collected 6 slices, resulting in a total EPI scan time of 5 m 30 s. 54 spiral timepoints (27 averages) were collected with the spiral scan to match the EPI scan time. Data were acquired with three free‐field pressure levels: 7, 4, and 2 MPa. The FDA mechanical safety limit is stated in terms of mechanical index (MI), defined as the peak negative pressure of the FUS pulse measured in free‐field water, derated by a depth attenuation factor and divided by the square root of the center frequency in MHz. The FDA MI limit for diagnostic ultrasound is 1.9. Assuming 39% transmission through the skull, the free‐field pressures used here correspond to 2.7, 1.6, and 0.8 MPa at the focus, and transcranial MIs (${\text{MI}}_{\text{tc}}$'s) of 3.4, 1.9, and 0.96, i.e., above, at, and below the FDA MI limit for diagnostic ultrasound. An additional spiral dataset with no ultrasound (0 MPa) was collected to assess displacement errors vs. number of averages.

A second set of single‐slice spiral experiments was performed with the same pressure levels as the EPI comparison, to compare alternating vs. previously reported blocked phase contrasts.^13^ The spiral scans were collected twice, once with the proposed alternated triggering for 100 time points (50 averages), and again with block triggering, in which 50 time points were collected consecutively with the positive phase contrast, and an additional 50 time points were collected with negative phase contrast. The total scan time for both was 1 m 42 s. Displacement maps were calculated from both scans using the model‐based method.

## 3 RESULTS

**FIGURE 3:**
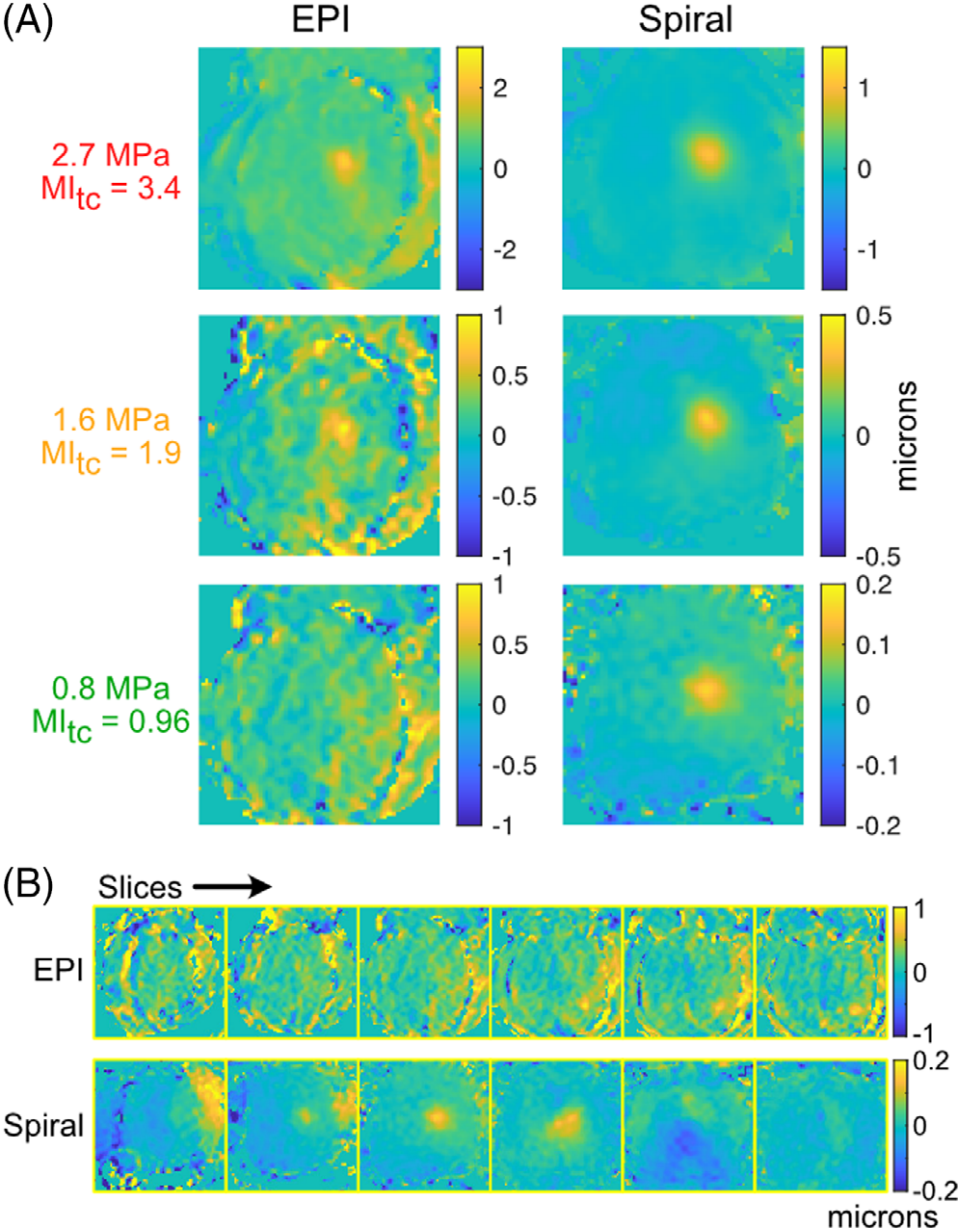
(A) EPI and spiral displacement maps for three pressures, with matched scan times. (B) 0.8 MPa EPI and spiral displacement maps across all six slices.

Figure 3A shows displacement maps centered in the focus in the slice dimension (third slice) for the EPI and spiral scans. The focus is clearly visible in the EPI maps above the MI limit, but becomes less visible at the MI limit, and disappears below it. The focus is clearly visible at all pressures in the spiral scan. The EPI maps further contain curved vertical artifacts that interfere with the focus. Figure 3B shows all the slices at 0.8 MPa, confirming that the focus is not visible in any EPI slice. Outside the focus in the brain, the EPI standard deviation was 143 nm, and the spiral standard deviation was 14.7 nm. The peak displacement in the high‐pressure EPI maps is larger than the spiral (2.39/1.00/(no visible focus) microns above/at/below the MI limit vs. 1.06/0.41/0.16 microns). This is partially explained by positive offsets in the nonfocal region in the EPI maps of 0.6 and 0.3 microns above and at the MI limit; such offsets are removed from the spiral maps by the model‐based reconstruction.

**FIGURE 4:**
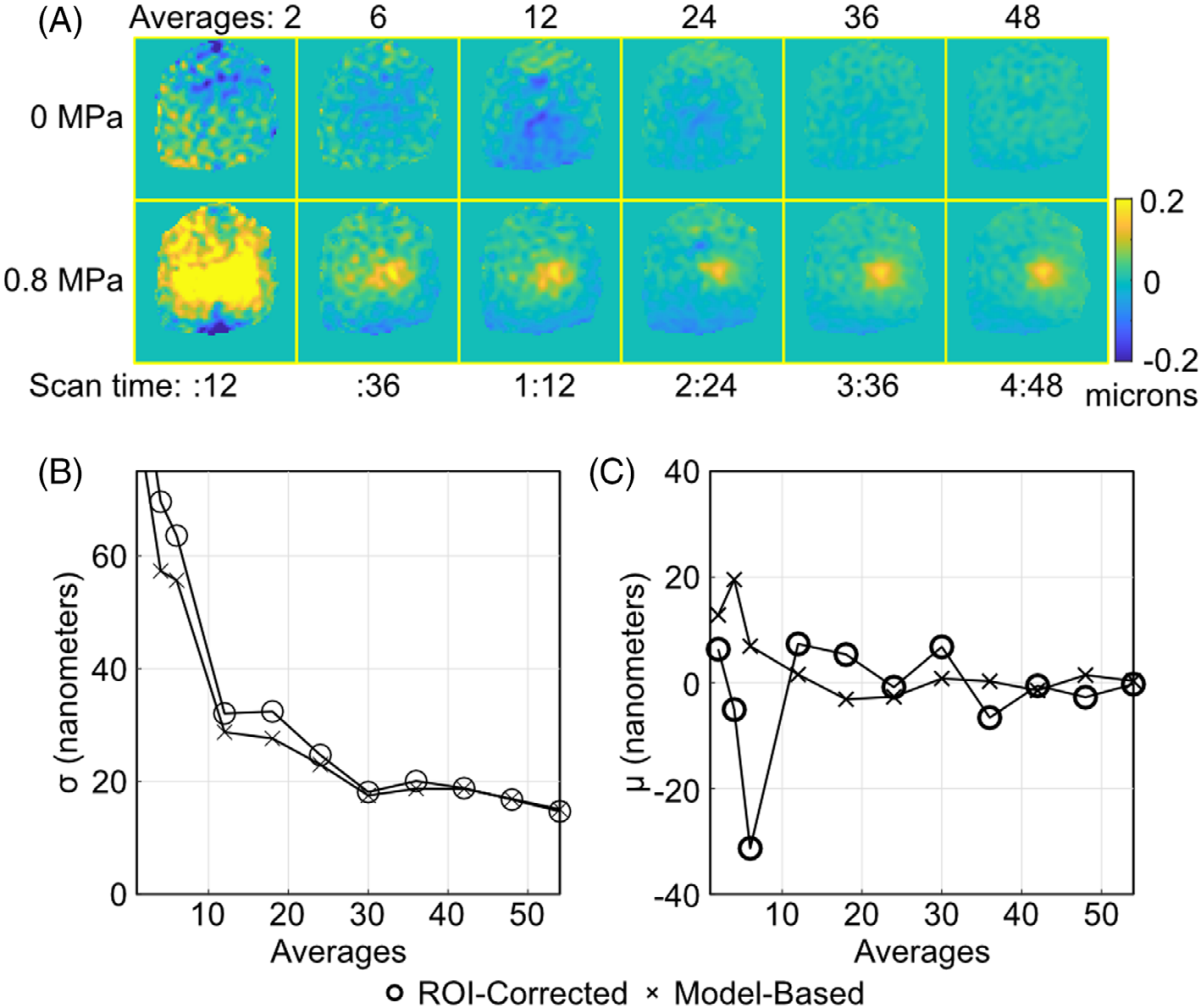
(A) Brain‐masked spiral displacement maps acquired with no ultrasound (0 MPa) and 0.8 MPa, vs. the number of averages used to compute the maps. Scan time is reported under each column for the six‐slice scan in mm:ss format. (B) 0 MPa displacement standard deviations vs. averages in the brain, for ROI‐corrected and model‐based maps. (C) 0 MPa displacement means vs. averages in the brain.

Figure 4A shows brain‐masked spiral displacement maps acquired with 0.8 MPa and no ultrasound, as a function of the number of averages, in the third slice of the six‐slice datasets. The focus is well‐visualized at 24 averages (2 m 24 s scan time), and further averaging principally serves to reduce background errors. The scan times reported below each column will be six times shorter for a single‐slice scan. Figure 4B plots out‐of‐focus displacement standard deviation vs. number of averages, and 4C plots mean out‐of‐focus displacement vs. number of averages, for ROI‐corrected and model‐based displacement map calculation. These plots were generated by repeating the displacement map calculation ten times for each number of averages, with randomly selected averages from the timeseries. The plots show that while model‐based calculation consistently improved displacement precision, thereby maximizing the benefit of averaging, it principally served to suppress large background errors: The standard deviations were 11.1% lower on average for the model‐based reconstruction, but the mean background errors were 30.5% lower in amplitude on average, and were more consistently close to zero for increasing averaging. Dividing a 160 nm peak displacement, as reported above for 0.8 MPa, by the standard deviations yields a contrast‐to‐noise ratio better than 5 for 12 averages and better than 10 for 48 averages.

**FIGURE 5:**
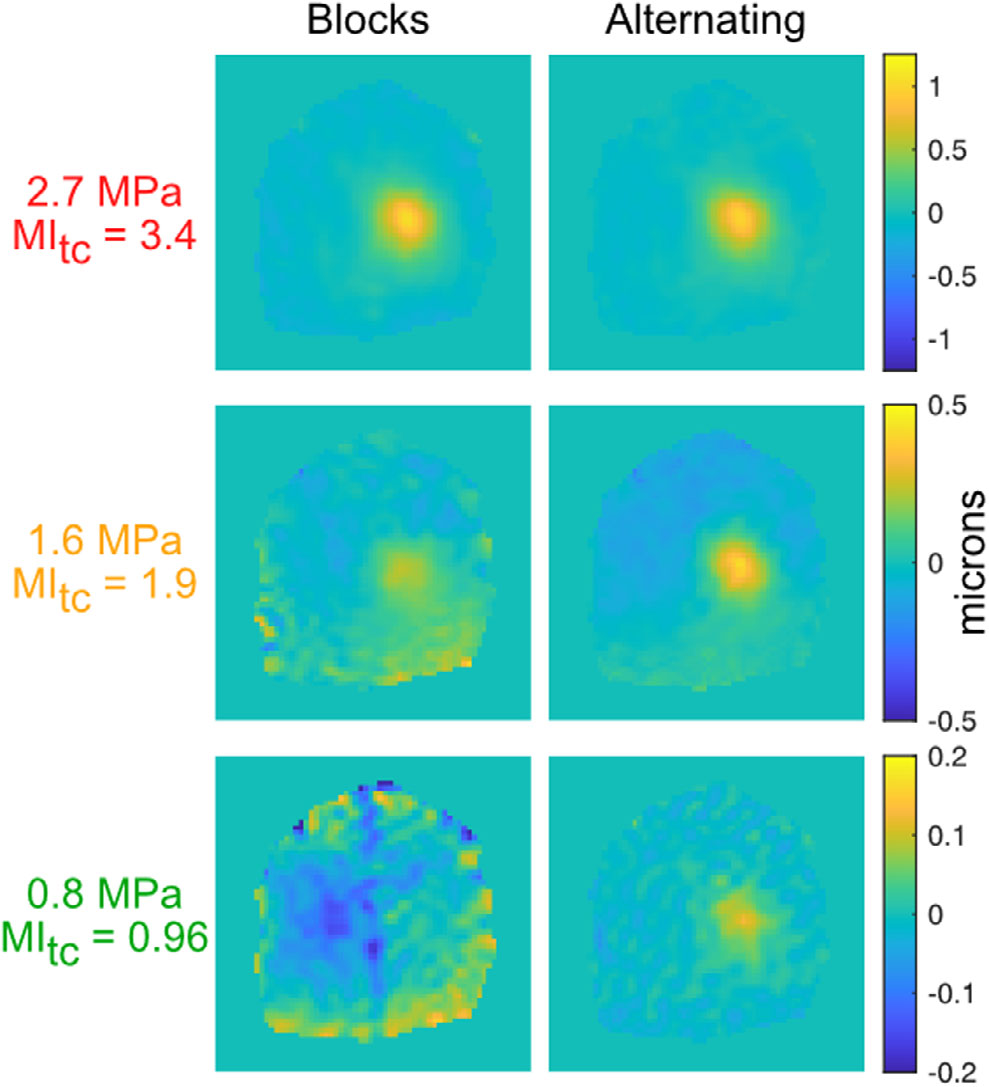
Blocked vs. alternating trigger displacement maps for three pressures, with 100 timepoints each.

Figure 5 shows single‐slice displacement maps for blocked‐ and alternating‐trigger spiral MR‐ARFI at three pressure levels. The focus is strong in the above‐ and at‐MI limit cases with alternating triggering, and weak but still visible in the below‐limit case. With block triggering, the focus is clearly visible at high pressure, is weak but still visible in the at‐MI limit case, and is invisible in the below‐limit case. Outside the focus in the brain, the blocked standard deviation was 66 nm at 0.8 MPa, and the alternating standard deviation was 13.4 nm.

## 4 DISCUSSION AND CONCLUSIONS

We have reported an alternating‐trigger high‐average single‐shot spiral MR‐ARFI pulse sequence and a model‐based displacement map calculation algorithm. By acquiring the two MR‐ARFI phase contrasts as close as possible in time, the sequence is inherently robust to dynamic sources of confounding phase errors such as respiration, and it was able to detect *in vivo* ultrasound foci with amplitudes between 100 and 200 nm. The model‐based displacement calculation suppressed the spatiotemporally varying phase errors, which stabilized the measurements as a function of the number of averages, and increased the benefit of averaging compared to an ROI‐based phase correction, without requiring manual selection of an ROI. These capabilities were demonstrated in comparison to a matched 3D spin‐echo EPI MR‐ARFI sequence previously implemented by our group, and in comparison to a blocked scheme used in the first reported high‐average spiral MR‐ARFI work.^13^ High‐average single‐shot spiral MR‐ARFI is related to the previous spiral MR‐ARFI work that has principally leveraged spiral readouts for rapid acquisition,^17^, ^18^ but uses a different sequence design to enable detection of very small nanometer‐level (rather than more typical micron‐level) displacements with robustness to physiological phase variations.

While our 3D EPI scan has provided clear focus visualization in the non‐human primate brain for several mechanistic and development studies,^9^, ^19^, ^20^, ^21^, ^22^ those studies used pressures above the MI limits, and we were motivated to improve MR‐ARFI's sensitivity to bring the pressure below MI limits to de‐risk its application in humans. More specifically, the present results demonstrated that phase artifacts and insufficient SNR in the 3D EPI scan prevented focus visualization below the MI limit, but the proposed spiral sequence and processing provided a clear visualization at half the MI limit, owing to its 10×‐better displacement precision. The EPI displacement maps contained large positive background offsets that increased focal displacement compared to the spiral maps, and could lead to incorrect analyses of delivered acoustic energy inferred from those displacements. The cause of these offsets requires study, but it is possible that the entire brain slice is displaced by the ultrasound pulse, as we have also observed it with the spiral scan, regardless of the trigger ordering (results not shown); however, it is removed by the zeroth‐order polynomial term in the model‐based displacement calculation. Since the primary use of MR‐ARFI in the brain is to determine and correct the focal position, a zero background is desirable to maximize the conspicuity of the focus. The offsets, however, only explain approximately half the difference between the EPI and spiral displacements. The remaining difference is likely due to blurring due to transverse relaxation and off‐resonance during the long (27.5 ms) spiral readout. These effects could be mitigated with the collection of a separate off‐resonance field map and off‐resonance‐corrected reconstruction,^23^ but this would require an offline reconstruction. They could also be mitigated with the use of a multi‐shot spiral readout. Like Reference,^13^ a single‐shot readout was used in this study to minimize sensitivity to physiological motion and phase variations between shots, which can easily overwhelm the very small displacement‐induced phase shifts (0.015 radians for 200 nm displacement). However, it may be possible to sufficiently suppress unwanted phase variations with the use of variable density sampling or separate navigators.

The FUS pulse repetition frequency was 1 Hz in this study to reduce the risk of thermal damage when sonicating at high pressure.^12^ This is especially important in the skull, where more heat can be deposited during transcranial FUS procedures.^19^ The lower pressures afforded by the spiral readout can further reduce thermal risk but can also allow for a higher pulse repetition rate, allowing for the scan to be performed in a shorter time, reducing overall therapy time.

The comparison to blocked phase contrasts showed that, by minimizing the average time between phase contrasts, alternating phase contrast is inherently more robust to dynamic phase errors arising from respiration and cardiac pulsation. The focus could not be visualized at the lowest pressure with blocked contrasts, but remained visible for alternating contrasts, and the out‐of‐focus displacement precision was 4× better for alternating contrasts. This result was obtained despite using the same model‐based displacement calculation for both sequences. Our implementation of the blocked sequence was very similar to that reported in Reference [13] with the exception of the MEG scheme: That work used 25% stronger 50 mT/m bipolar MEGs with longer total durations of 20 or 30 ms (vs. the 14 ms total duration used here), and ultrasound was switched on and off to generate the two‐phase contrasts. The primary difference from that work was our displacement map calculation. The processing in Reference [13] applied an fMRI‐like correction pipeline and modeling to the displacement maps, motivating the use of blocked phase contrasts. It started with physiologic correction to remove respiratory and cardiac signal variations, then applied general linear modeling with bulk phase and quadratic nuisance terms, and ended with high‐pass filtering to remove spatially smooth errors. This processing and the model‐based reconstruction applied here address the same effects, since both remove spatiotemporally varying phase shifts that are unrelated to tissue displacement. It is possible that, via its independent temporal filtering of each voxel's signal, the processing in Reference [13] would better suppress unwanted phases that are spatially high‐frequency in nature, such as pulsating blood vessels. While the model‐based method also removes unwanted temporally varying signals, it assumes that those signals are spatially smooth, so it would not be able to remove sharp pulsating features. Nevertheless, we preferred the model‐based approach for its ability to accommodate arbitrary phase contrast schedules and because it generates quantitative displacement maps in spatial units. The displacement maps could be independently verified using methods such as ultrasound‐ARFI.^24^

## CONFLICT OF INTEREST STATEMENT

William A. Grissom holds equity in and is a contractor for Nudge Workbench, LLC.

## Data Availability

Code and an example dataset for the method are available at https://github.com/grissomlab/modelBasedSpiralARFI. For this work, the version with SHA‐1 hash ee64b631f19af6b2ce634f5a99ae43ce03a73edd was used.
